
JOURNAL INFORMATION
==============================
NLM Title Abbreviation: BJPsych Bull
Journal ID: BJPsych Bull
NLM Journal ID: 101650950
Journal ID: BJB

BJPsych Bulletin

ISSN: 2056-4694
EISSN: 2056-4708
Publisher: Royal College of Psychiatrists

ARTICLE INFORMATION
==============================
PMCID: PMC12314402
DOI: 10.1192/bjb.2025.10131
Article ID: S2056469425101319
Article version: 1
Subjects: Front Cover (OFC, IFC) and matter

BJB volume 49 issue 4 Cover and Front matter

Print publication date: 2025 Aug
Volume: 49
Issue: 4
First page: f1
Last page: f2
Copyright: © The Author(s) 2025
Copyright year: 2025
Copyright holder: The Author(s)
License: This is an Open Access article, distributed under the terms of the Creative Commons Attribution licence (http://creativecommons.org/licenses/by/4.0/), which permits unrestricted re-use, distribution, and reproduction in any medium, provided the original work is properly cited.
License URL: https://creativecommons.org/licenses/by/4.0/

Page count: 2

==============================

AUGUST 2025 VOL 49 ISSUE 4

### BJPsych Bulletin

RC PSYCH
ROYAL COLLEGE OF PSYCHIATRISTS

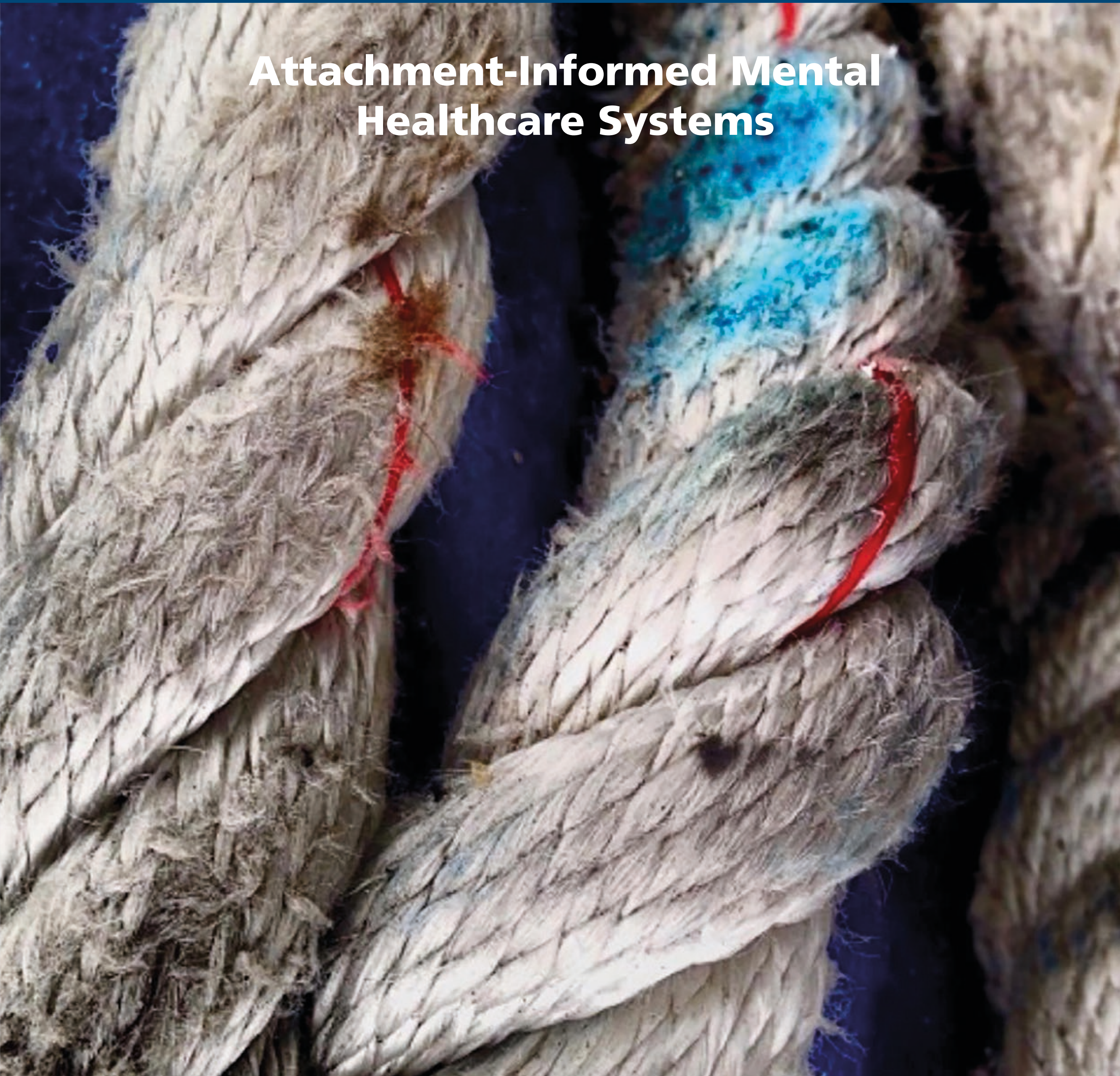

### Attachment-Informed Mental Healthcare Systems

BJPsych
### Bulletin

**Editor**
Andrew Forrester

**Associate Editors**
Neil Armstrong (UK)
Catherine Penny (UK)

**Editorial Board**
Niruj Agrawal (UK)
Lauren Atkinson (UK)
Oyedeji Ayonrinde (Canada)
Cate Bailey (UK)
Sally Barlow (UK)
Niall Galbraith (UK)
Sanju George (India)
Philip Graham (UK)
Andrew Hadler (UK)
Neel Halder (UK)
Natasha Kalebic (UK)
Justin Marley (UK)
Rob Poole (UK)
Lucy Potter (UK)
Gopinath Ranjith (UK)
Louise Robinson (UK)
Harriet Stewart (UK)
Kai Syng Tan (UK)
Peter Tyrer (UK)
Lauren Waterman (UK)
Mei Cheung Wong (Hong Kong)

**Book Reviews Editors**
Allan Beveridge (UK)
Femi Oyebode (UK)

**Correspondence Editor**
William Badenhorst (UK)

**Culture Editor**
David Foreman (UK)

**Drugs and Therapeutics Editor**
Nitin Gupta (India)

**Education & Training Handling Editor**
Rahul Bhattacharya (UK)

**Interview Editor**
Abdi Sanati (UK)

**Obituaries Editor**
Claire Hilton (UK)

**Qualitative Research Editor**
Joanne Lloyd (UK)

**Visual Images Editor**
Alex Acosta-Armas (UK)

**Managing Editor**
Conceição Santos Sinclair

**Notice to contributors**
Please refer to our online instructions for manuscript preparation and submission at https://www.cambridge.org/core/journals/bjpsych-bulletin/information/instructions-contributors

**Subscriptions**
*BJPsych Bulletin* is Open Access online. For print subscriptions, contact Cambridge University Press and Assessment, Journals Fulfilment Department, Shaftesbury Road, Cambridge CB2 8EA, UK, email journals@cambridge.org; or in the USA, Canada and Mexico, to Cambridge University Press, Journals Fulfilment Department, 100 Brook Hill Drive, West Nyack, New York 10994–2133, email subscriptions_newyork@cambridge.org. Japanese prices for institutions are available from Kinokuniya Company Ltd, P.O. Box 55, Chitose, Tokyo 156, Japan.

Print-only subscription rate for 2025: £122/$194

Periodicals postage paid at New York, NY, and at additional mailing offices. POSTMASTER: Send address changes in USA, Canada and Mexico to: BJPsych Bulletin, Cambridge University Press, Journals Fulfilment Department, 1 Liberty Plaza, Floor 20, New York, NY 10006, USA.

Queries from College members about missing or faulty print copies should be addressed to Customer Services, Cambridge University Press, email journals_societies@cambridge.org, tel. +44 (0)1223 326070.

**Publication information, editorial policy, submissions**
*BJPsych Bulletin* is published bimonthly (6 issues per year) on behalf of the Royal College of Psychiatrists, a charity registered in England and Wales (228636) and in Scotland (SC038369).

Manuscripts for publication should be submitted online at https://mc.manuscript central.com/bjpsych-bulletin

*BJPsych Bulletin* is hosted on the Cambridge Core service at https://www.cambridge.org/core/journals/bjpsych-bulletin

Information about the College's publications is available at https://www.rcpsych.ac.uk

All papers in this journal are peer-reviewed. No person is permitted to take any role in the peer-review of a paper in which they have an interest, defined as follows: fees or grants from, employment by, consultancy for, shared ownership in, or any close relationship with, an organisation whose interests, financial or otherwise, may be affected by the publication of the paper.

*BJPsych Bulletin* complies with the World Association of Medical Editors' guidelines on editorial independence (http://www.wame.org/policy-statements#Relationship between Editors and Owners) and with the Committee on Publication Ethics' guidelines on best publication practice (https://publicationethics.org/resources/guidelines).

Formerly published as *The Psychiatric Bulletin* (2014), ISSN 2053-4868; *The Psychiatrist* (2010-2013), ISSN 1758-3209; *Psychiatric Bulletin* (1988-2009), ISSN 0955-6036; and *Bulletin of the Royal College of Psychiatrists* (1977-1988), ISSN 0140-0789.

Printed in Great Britain by Henry Ling Limited, The Dorset Press, Dorchester, Dorset DT1 1HD.

For EU product safety concerns, contact us at Calle de José Abascal, 56, 1°, 28003 Madrid, Spain, or email eugpsr@cambridge.org.

This journal issue has been printed on FSC™-certified paper and cover board. FSC is an independent, non-governmental, not-for-profit organization established to promote the responsible management of the world's forests. Please see www.fsc.org for information.

FSC www.fsc.org MIX Paper | Supporting responsible forestry FSC™ C013985

Print ISSN: 2056-4694
Online ISSN: 2056-4708

INDEXED IN EMERGING SOURCES CITATION INDEX THOMSON REUTERS
